# Eosinophilic granulomatous polyangiitis with central nervous system involvement in children: a case report and literature review

**DOI:** 10.3389/fimmu.2024.1406424

**Published:** 2024-05-15

**Authors:** Nana Nie, Lin Liu, Cui Bai, Dahai Wang, Shan Gao, Jia Liu, Ranran Zhang, Yi Lin, Qiuye Zhang, Hong Chang

**Affiliations:** Department of Pediatric Nephrology, Rheumatology, and Immunology, The Affiliated Hospital of Qingdao University, Qingdao, China

**Keywords:** eosinophilic granulomatosis with polyangiitis, child, central nervous system, diagnosis, therapy

## Abstract

**Objective:**

To explore the clinical characteristics and treatment outcomes of children with central nervous system (CNS) involvement in eosinophilic granulomatosis with polyangiitis (EGPA).

**Methods:**

A child who presented with EGPA complicated by CNS involvement was admitted to our hospital in June 2023. The clinical features were analyzed retrospectively, and relevant literatures were reviewed to provide a comprehensive overview of this condition.

**Results:**

A ten-year-old girl, who had a history of recurrent cough and asthma accompanied by peripheral blood eosinophilia for eight months, was admitted to our hospital. On admission, spotted papules were visible on her hands and feet, bilateral pulmonary rales were audible. The laboratory examination revealed that the proportion of eosinophils (EOS) exceeded 10% of white blood cells, the anti-neutrophil cytoplasmic antibody (MPO-ANCA) was positive, the immunoglobulin G level was 15.80g/L, and the immunoglobulin E level was greater than 2500.00IU/mL. The imaging examination showed multiple patchy and nodular high-density shadows in both lungs as well as sinusitis. Pulmonary function tests indicated moderate ventilation and diffusion dysfunction. Bone marrow cytology demonstrated a significant increase in the proportion of eosinophils. Skin pathology confirmed leukocytoclastic vasculitis. During the hospitalization, the child had a convulsion. The magnetic resonance imaging (MRI) scan of the brain showed multiple abnormal signal shadows in the bilateral cerebral cortex and the electroencephalogram (EEG) showed epileptic waves. Following the administration of methylprednisolone pulse therapy in combination with cyclophosphamide treatment, her cough and asthma resolved, the skin rash disappeared without any further convulsions. We found that only a young EGPA patient with CNS involvement had been previously reported. The previously reported case began with long-term fever, weight loss, and purpuric rash. Both patients responded well to treatment with glucocorticoids and cyclophosphamide, experiencing significant improvement in their clinical symptoms and normalization of their peripheral blood eosinophils.

**Conclusion:**

The diagnosis of EGPA in children can be challenging. When a child is affected by EGPA, it is essential to remain vigilant for signs of CNS involvement. The treatment with glucocorticoids and cyclophosphamide is effective in managing EGPA in children.

## 1 Introduction

Eosinophilic granulomatosis with polyangiitis is a rare autoimmune vasculitis that primarily affects small- to medium-sized blood vessels (1). The onset of EGPA is generally observed between the ages of 25 and 50, with a reported prevalence among adults ranging from 10 to 13 per million. Nevertheless, EGPA can also manifest during childhood, carrying a notable attendant morbidity and mortality (2). Characterized by eosinophilia, granulomatous inflammation, and necrotizing vasculitis, it typically manifests with a wide range of symptoms including asthma, eosinophilia, and systemic vasculitis. Furthermore, it has the potential to affect numerous organs, including the kidneys, heart, digestive tract, and nervous system. Notably, in EGPA, peripheral nervous system involvement is more prevalent, whereas central nervous system involvement occurs less frequently. The pathophysiology of EGPA involves the dysregulation of the immune system, specifically the activation of eosinophils, which trigger inflammation and tissue damage in affected organs. Recent studies have unequivocally established the significance of ANCA in tissue damage from a clinicopathological perspective (2). The diagnosis of the disease relies on a combination of clinical findings, laboratory tests, and histopathological examination. Treatment typically involves the use of immunosuppressive drugs and corticosteroids, aimed at suppressing the overactive immune system and reducing inflammation (3). In this study, we retrospectively analyzed the clinical characteristics of a pediatric case of EGPA complicated by CNS involvement.

## 2 Case presentations

A ten-year-old girl, who had a history of recurrent cough and asthma accompanied by peripheral blood eosinophilia for eight months, was admitted to our hospital in June 2023. In January and March 2023, she was hospitalized at another hospital due to recurrent cough and asthma. During the period, multiple blood tests revealed a significant increase in eosinophil count and IgE levels. MPO-ANCA was positive while the antinuclear antibodies profile was negative. Allergen testing confirmed strong sensitization to dust mites. Chest CT scan reveals augmented thickness and density of the pulmonary trachea, numerous ground-glass opacities and nodular shadows scattered throughout the lungs, as well as enhanced bronchial wall thickness. Nasal sinus CT scan revealed acute sinusitis. Pulmonary function test indicated moderate ventilation and diffusion dysfunction. Bone marrow cytology showed hyperplastic bone marrow with a notable increase in eosinophil proportion. Lung alveolar lavage fluid examination did not detect pathogenic bacteria. Genetic testing through whole exome sequencing did not identify any pathogenic mutations associated with the clinical phenotype. During the second hospitalization, a transient red rash appeared on her left wrist. Following treatment with anti-infective agents, glucocorticoids, nebulization, her eosinophil count normalized and the rash disappeared. After discharge, she was prescribed oral prednisone acetate, which was gradually tapered.

Eleven days prior to admission, she experienced unprovoked intermittent coughing, accompanied by yellowish and purulent sputum. The cough, which was particularly severe during the night, was accompanied by wheezing and dyspnea. Additional symptoms included nasal congestion and yellowish loose stools. Notably, the eosinophil count in the blood routine examination at another hospital was once again elevated. Despite treatment with azithromycin for anti-infection, the child’s condition did not improve. Five days prior to admission, she developed a fever accompanied by red rashes on the extensor surfaces of the feet and hands. The cough intensified, and there was hemoptysis, which was pinkish and streaky in nature. The hemoptysis episodes occurred at regular intervals of 10–20 minutes. The child had previously been in excellent health and had no significant personal history. Her father had a history of allergic rhinitis, and her mother had an allergic constitution.

**Figure 1:**
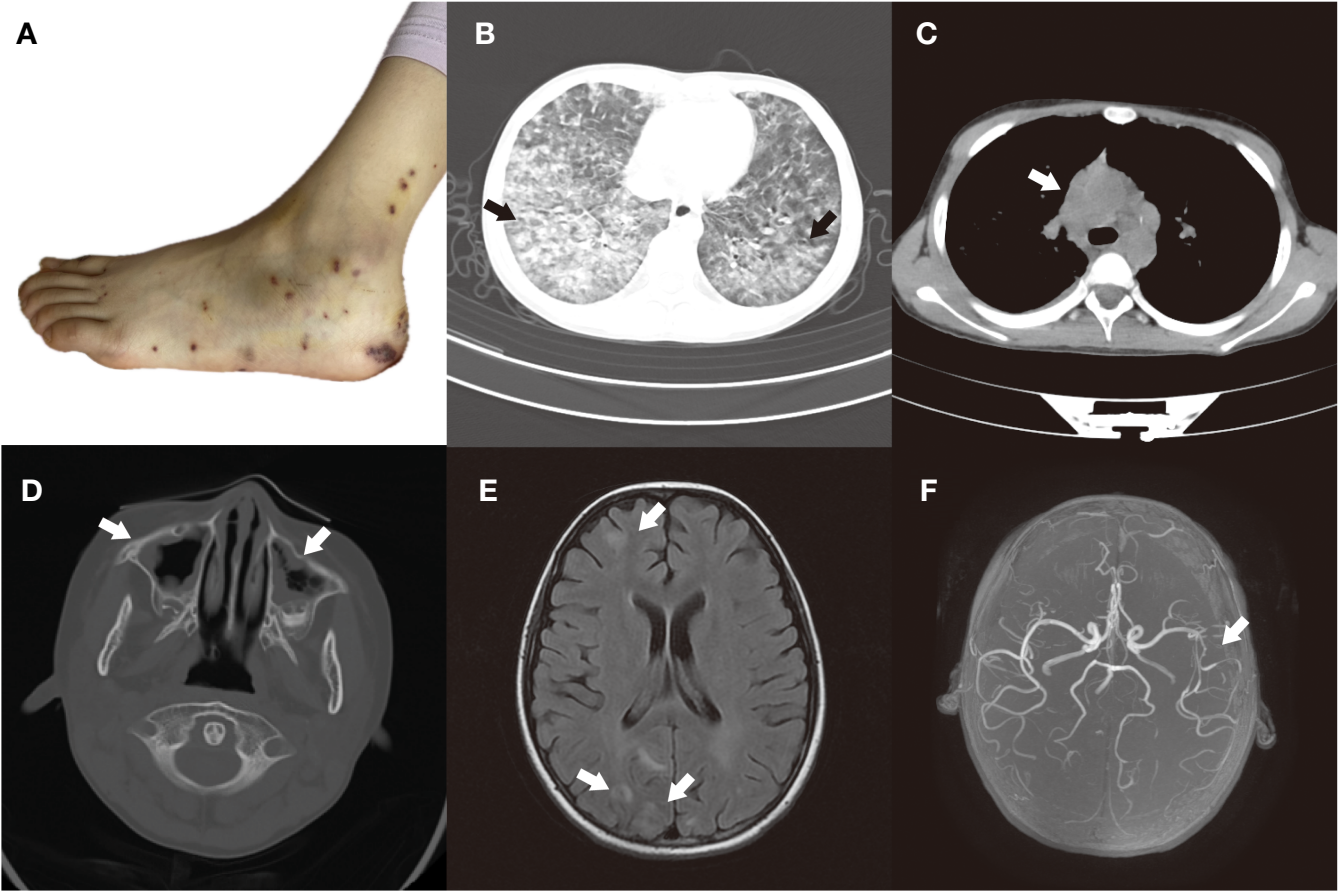
**(A)** Foot rash, partially ulcerated and scabbed. **(B, C)** Before treatment, the HRCT scan of the chest showed multiple patchy and nodular lesions with unclear boundaries, indicating high-density shadows in both lungs. And multiple enlarged lymph nodes were observed in the mediastinum and bilateral hilar regions. **(D)** Before treatment, the CT plain scan of the paranasal sinuses revealed a notable thickening of the mucosa in the bilateral maxillary, ethmoid, sphenoid, and frontal sinuses. **(E)** Before treatment, the MRI scan of the brain showed multiple abnormal signal shadows in the bilateral cerebral cortex. **(F)** Before treatment, the MRA revealed moderate to severe stenosis in the middle cerebral artery segment M2 of the left middle cerebral artery.

On admission, she revealed a weight of 29kg (P25-P50), a height of 146cm (P75-P90), a temperature of 36.2°C, a heart rate of 135 beats per minute, a respiratory rate of 40 beats per minute, the blood pressure of 95/60mmHg, and the oxygen saturation (SP02) ranging between 93% and 94%. She was conscious but in a poor mental state. Spotted papules were visible on her hands and feet, which were partially ulcerated and scabbed, accompanied by pain (**Figure 1A**). Additionally, pulmonary rales were audible bilaterally. The abdomen was soft, with a 2cm indentation under the right rib cage of the liver. The cardiac and neurologic examinations were unremarkable.

The laboratory tests showed white blood cell count of 13.66×10^9^/L (4.3×10^9^-11.3×10^9^), lymphocyte count of 1.27×10^9^/L (1.5×10^9^-4.6×10^9^), eosinophil count of 4.11×10^9^/L (0.00×10^9^-0.68×10^9^), platelet count of 314×10^9^/L (167×10^9^-453×10^9^), hemoglobin of 118g/L (118–156), C-reactive protein of 67.68mg/L (0–5); erythrocyte sedimentation rate of 43.00mm/60min (0–20), procalcitonin of 0.181ng/mL (<0.05), albumin of 23.5g/L (39–54), D-dimer of 7310ng/mL (0–500), ferritin of 438ng/ml (13–84). The blood culture and sputum culture were negative, and the blood coagulation, liver function, kidney function, myocardial enzymes, urine, and stool samples were all within normal limits. The immunological assessment revealed that the immunoglobulin G level was 15.80g/L, while the immunoglobulin E level was significantly elevated at >2500.00IU/mL. Detailed lymphocyte subset analysis reveals lymphocytes constitute 3.98% (11.4%-57%) of white blood cells, and among lymphocytes, T lymphocytes represent 63.71% (53.7%-82.8%). Helper T lymphocytes, CD4+ naive T cells and CD4+ effector T cells account for 60.55% (46.2%-78%), 26.39% (7.2%-68.9%), 24.56% (0.44%-6.06%) of total T lymphocytes, respectively. Plasmablasts represent 0.29% (1.9%-23.7%) of B lymphocytes. Other lymphocyte subsets such as NK cells, CD4+ central memory T cells, regulatory T cells, CD8+ T cells, double negative T cells, B lymphocytes, and dendritic cells are generally normal. The tests for antinuclear antibody, complement, ASO and RF were all negative. The aetiological examination indicated that the five respiratory pathogens, Mycoplasma pneumoniae, fungal G test, fungal GM test, and tuberculosis infection T cell were all negative.

The high-resolution chest computed tomography (HRCT) imaging revealed multiple patchy and nodular lesions with unclear boundaries, indicating high-density shadows in both lungs (**Figure 1B**). Additionally, multiple enlarged lymph nodes were observed in the mediastinum and bilateral hilar regions (**Figure 1C**), and a small amount of pleural effusion was present on the right side. The CT plain scan of the paranasal sinuses revealed a notable thickening of the mucosa in the bilateral maxillary, ethmoid, sphenoid, and frontal sinuses (**Figure 1D**). Within the sinus cavities, there were scattered patches of low-density lesions, and no significant signs of bone destruction were detected in the sinus walls. Pulmonary function tests indicated moderate ventilation and diffusion dysfunction. Bone marrow cytology demonstrated a significant increase in the proportion of eosinophils. Electrocardiogram displayed sinus rhythm and irregular sinus rhythm. Echocardiogram revealed mild mitral and tricuspid valve regurgitation.

After admission, the child was given piperacillin sodium and tazobactam sodium for anti-infection, nebulized for cough and asthma. However, on the second day of hospitalization, she continued to experience fever, coughing, hemoptysis, and labored breathing. Even under oxygen inhalation, her oxygenation remained inadequate. Therefore, the child was transferred to the Pediatric Intensive Care Unit (PICU) for tracheal intubation and ventilator support treatment under sedation and analgesia. Meanwhile, meropenem was upgraded for anti-infection, etamsylate and hemocoagulase were given for hemostasis.

**Figure 2:**
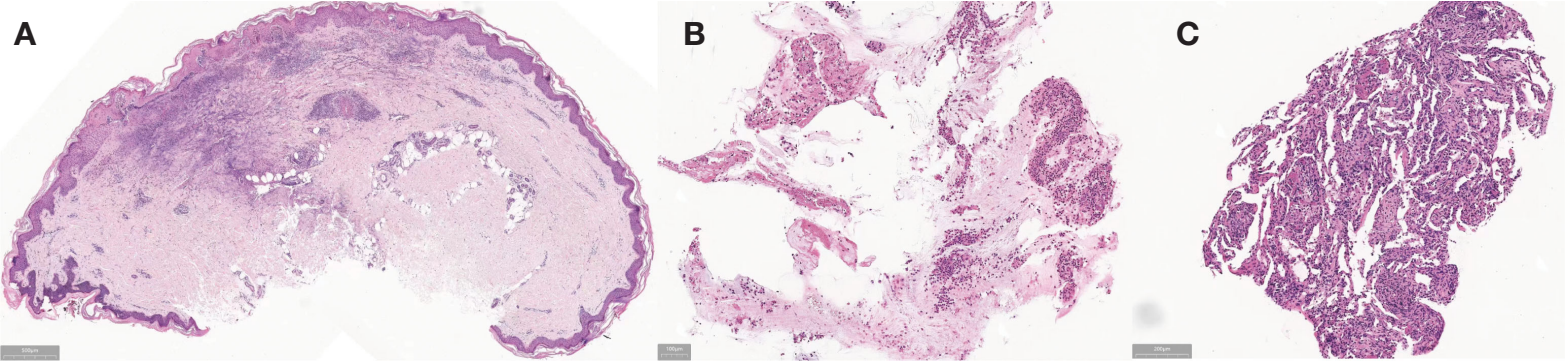
**(A)** Before treatment, the skin pathological examination of the left ankle showed a reticulated pattern of hyperkeratosis, accompanied by necrosis in the central epidermis. In the superficial and middle layers of the dermis, fibrinoid necrosis is observed in the blood vessel walls, concurrent with neutrophil infiltration. Numerous neutrophils and eosinophils are visible infiltrating the areas surrounding the blood vessels, while erythrocyte extravasation and nuclear dust are also apparent. Notably, no distinct granulomatous structure is evident, and the overall changes are indicative of leukocytoclastic vasculitis. **(B, C)** Before treatment, the pathological examination of the lung tissue biopsy indicated the presence of chronic suppurative inflammation, accompanied by fibrous tissue proliferation and fibrosis.

Then she underwent bronchoscopy, revealing significant inflammatory and nodular changes in the bronchial mucosa under microscopic examination. Lung lavage fluid metagenomic test came back negative, while the culture suggested EB virus infection. Cytological classification showed neutrophils at 23.00% (<2%) and eosinophils at 13.00% (<1%). Therefore, we enhanced the etiological examination of EB. The EB virus capsid antigen IgM was 8.19AU/mL (<3), and EB virus DNA was 2.03e+003 (≤5.0e+003). Subsequently, we added ganciclovir antiviral therapy to the treatment plan. Furthermore, the skin pathological examination of the left ankle showed a reticulated pattern of hyperkeratosis, accompanied by necrosis in the central epidermis. In the superficial and middle layers of the dermis, fibrinoid necrosis is observed in the blood vessel walls, concurrent with neutrophil infiltration. Numerous neutrophils and eosinophils are visible infiltrating the areas surrounding the blood vessels, while erythrocyte extravasation and nuclear dust are also apparent. Notably, no distinct granulomatous structure is evident, and the overall changes are indicative of leukocytoclastic vasculitis (**Figure 2A**). The pathological examination of the lung tissue biopsy indicated the presence of chronic suppurative inflammation, accompanied by fibrous tissue proliferation and fibrosis (**Figures 2B, C**).

The patient, a school-age girl, has a known history of recurrent asthma. Over the course of the illness, she has developed skin and lung involvement. Her peripheral blood eosinophil count has repeatedly been found to be more than 10%, and a sinus CT scan has demonstrated sinusitis. After using the clinical diagnostic criteria established by the American College of Rheumatology in 1990 (4), we confirmed the diagnosis of EGPA. After obtaining a clear diagnosis, we administered a methylprednisolone pulse therapy (500mg for 3 days followed by 750mg for 2 days) and cyclophosphamide (300mg for 2 days) to effectively treat the condition. In addition, we administered piperacillin tazobactam to combat infections. Following these treatments, her temperature normalized, coughing, wheezing, and hemoptysis symptoms resolved, and the rash showed signs of improvement.

On the seventh day of hospitalization, the child had a convulsion once, with both eyes exhibiting an upward gaze, and a blood pressure of 125/72mmHg. The child’s consciousness remains unknown while sedated. The EEG showed epileptic waves. We have perfected the lumbar puncture procedure and did not detect any abnormalities in the cerebrospinal fluid routine, biochemistry, immunoglobulin, bacterial culture, and smear. MRI scan of the brain showed multiple FLAIR hyperintensities in the bilateral cerebral cortex (**Figure 1E**). The magnetic resonance angiography (MRA) revealed moderate to severe stenosis in the middle cerebral artery segment M2 of the left middle cerebral artery (**Figure 1F**), while the magnetic resonance venography (MRV) showed no significant abnormalities. To control the epileptic seizures, she was administered levetiracetam. After treatment, the child no longer experienced seizures.

**Figure 3:**
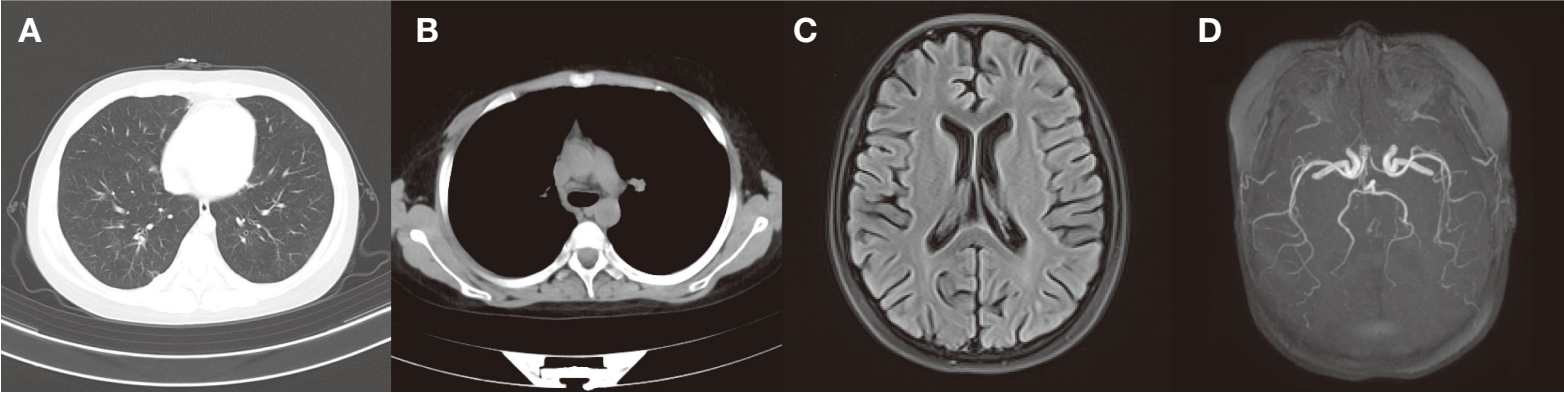
**(A, B)** After treatment, the chest CT scans revealed significant improvement in lung inflammation and no obvious enlarged lymph nodes were seen in the mediastinum and bilateral hilar. **(C)** After treatment, the cranial MRI showed a reduction in the abnormal signal intensity in the bilateral cerebral cortex compared to the previous scan. **(D)** After treatment, there were no markedly abnormal findings in cranial MRA.

At the follow-up review conducted one month following discharge, her peripheral blood eosinophil count returned to normal levels. Chest CT scans revealed significant improvement in lung inflammation (**Figures 3A, B**), pulmonary function test showed mild ventilation-diffusion dysfunction. And the cranial MRI showed a reduction in the abnormal signal intensity in the bilateral cerebral cortex compared to the previous scan (**Figure 3C**) while the cranial MRA was unremarkable (**Figure 3D**). The patient continues to visit the outpatient department for regular follow-up appointments, adjust the dosage and frequency of drug administration based on laboratory and imaging test results. Currently, she is receiving maintenance treatment with 6 rounds of monthly intravenous cyclophosphamide already completed.

Using the search terms “eosinophilic granulomatosis with polyangiitis (EGPA)/Churg-Strauss syndrome”, “central nervous system” and “child” to search the China National Knowledge Infrastructure (CNKI), Wanfang database, and Chinese Biomedical Literature Database (up to December 2023), no reports of children were found. All reported cases were adults and the common clinical manifestations of the CNS were cerebral infarction or ischemia, subarachnoid hemorrhage, and cerebral hemorrhage. When we searched the PubMed database using the same search terms, we retrieved one relevant case (5). The patient, a 14-year-old boy, presented with a protracted fever, weight loss, muscle pain, joint pain, and a purpuric rash. He subsequently developed finger numbness, sinusitis, testicular pain, lung infiltration, asthma, and pericardial effusion. Laboratory tests revealed that eosinophils accounted for 58% of his peripheral blood count. The skin biopsy results indicated eosinophilic granulocytic infiltration necrotizing vasculitis. Following initial treatment with glucocorticoids and cyclophosphamide, the patient’s condition improved. However, during the course of his illness, he developed cerebral vasculitis secondary to epilepsy, and ultimately, hypoxemia and cardiac arrest triggered by severe asthma led to his demise. The case we reported is the first known instance of pediatric CNS involvement in China to date.

## 3 Discussion

EGPA is a multifaceted disease characterized by chronic rhinosinusitis, chronic sinusitis, asthma, and elevated levels of eosinophils in the peripheral blood. It was first described by pathologists J.Churg and L.Strauss in 1951, also known as the Churg-Strauss syndrome. EGPA is a rare disorder, with an annual incidence rate ranging from 0.5 to 6.8 per million individuals (6). It can affect individuals of all ages, with the median age of onset typically ranging from 38 to 54. There are no significant racial, familial, or gender disparities associated with EGPA. Additionally, few cases have been reported in children (7).

The cause of EGPA remains unknown, but it is thought to be associated with environmental and genetic factors. The primary mechanism behind the development of EGPA is caused by EOS infiltration and ANCA-induced damage to the vascular endothelium. Notably, approximately 70% of patients have a history of allergic rhinitis, and the onset often linked to asthma. Comprehensive examinations reveal elevated levels of EOS in the peripheral blood and tissues, along with increased IgE levels, indicating that EGPA may be a form of allergic or hypersensitivity disease (8).

EGPA is distinguished by eosinophilic infiltration and vasculitis, presenting with a diverse array of vascular-related clinical symptoms. Vasculitis primarily targets small-to-medium-sized vessels, resulting in a spectrum of issues linked to blood vessel dysfunction. Respiratory involvement, particularly affecting the lungs, is a prominent feature of EGPA. Additionally, patients may exhibit cutaneous vasculitis due to inflammation and necrosis in skin vessels. Cardiovascular, gastrointestinal, and nervous system involvements are also possible (2). A significant proportion of EGPA patients, up to 75%, experience peripheral neuropathy, often manifesting as polyneuropathy. In some instances, cranial nerves may also be implicated, though central nervous system involvement remains comparatively uncommon. And in a recent systematic analysis of 33 children with EGPA by Zwerina, it was observed that female patients were in the majority (9). Notably, when compared to adults, children tend to have more severe involvement of the heart and lungs, while the peripheral nerves and skeletal muscles are less.

The clinical features of EGPA are typically divided into three distinct stages: the prodromal phase, the vasculitis phase, and the extravascular granuloma phase. During the prodromal phase, patients typically exhibit atopic diseases with eosinophilia being most prevalent in the peripheral blood, lung and gastrointestinal tract. The vasculitis phase is characterized by the presence of systemic small-to-medium vessel vasculitis. Skin involvement is a frequent feature of this phase, affecting between 1/2 and 2/3 of EGPA patients. This involvement typically manifests as tender subcutaneous nodules on the extensor surfaces of the arms, hands and legs, as well as petechial or ecchymotic skin lesions. The heart is also often observed during this phase and represents one of the severe manifestations. The extravascular granuloma phase is the final stage, during which patients may develop granulomatous lesions in the nose, lungs, and intestines.

The diagnostic criteria for EGPA primarily rely on the clinical diagnostic criteria established by the American College of Rheumatology in 1990 (4). It includes: (1) a history of asthma (2) peripheral blood eosinophilia with a proportion exceeding 10% of white blood cells (3) involvement of paranasal sinuses (4) transient pulmonary infiltrates (5) neuropathy affecting single or multiple nerves (6) pathological confirmation of extravascular eosinophil infiltration. A patient who meets four or more could be diagnosed with EGPA. The lung and the skin are most commonly affected while EGPA can impact any system, including the cardiovascular, gastrointestinal, renal, and central nervous system. In the case we are reporting, the patient demonstrated involvement of the lung, skin and CNS during the course of the illness. Her peripheral blood eosinophil counts repeatedly exceeded 10%, and cranial imaging indicated sinusitis, fulfilling the diagnostic criteria. The time from onset to diagnosis was 8 months, exhibiting characteristics such as recurrent illness, multiple affected organs, and a severe condition compared to many adult EGPA cases.

The involvement of the CNS in EGPA is characterized by clinical manifestations that cannot be attributed to metabolic encephalopathy or other causes. It is estimated that approximately 70% of adult EGPA patients experience neurological involvement, although the CNS is less commonly affected (10). The involvement of the CNS mainly manifests as ischemic lesions, intracranial hemorrhage or subarachnoid hemorrhage, cranial nerve palsy and vision loss (11). The pathogenesis may be associated with secondary cerebral vasculitis and eosinophilic infiltration. Suying Liu et al. conducted a study on 110 EGPA patients aged between 19 and 80, and found that 17.3% had involvement of the CNS. The most common manifestation was ischemic lesions (63.2%), followed by reversible posterior encephalopathy syndrome (PRES) (36.8%) (12). And age, disease duration, and fever are potential independent risk factors for CNS involvement.

EGPA-associated PRES is characterized by patients presenting with typical acute neurological symptoms, along with radiological abnormalities of reversible vasogenic brain edema. This condition is often accompanied by seizures, encephalopathy, headache, and visual disturbances in the context of EGPA. The precise mechanism of PRES remains unclear, and it may be caused by changes in brain autoregulation due to the involvement of two mechanisms: hypertension and endothelial injury (13, 14). During the course of the patient’s illness, seizures occurred, blood pressure increased significantly compared to when the patient was admitted, cranial MR imaging demonstrated PRES manifestations, and MRA revealed cerebral artery stenosis. Consequently, a comprehensive evaluation encompassing the child’s clinical presentation, radiological findings, and response to therapy led to the conclusion that the patient was suffering from EGPA complicated by CNS involvement, manifesting as PRES and cerebrovascular stenosis.

At present, there are no confirmed laboratory indicators for EGPA. Some studies have shown that adults with EGPA who are ANCA-positive are more likely to exhibit clinical manifestations such as weight loss, ear, nose, and throat lesions, as well as peripheral neuropathy. While ANCA-negative individuals are more prone to tissue damage in the heart and lungs. In this particular case, the child was ANCA-positive and presented with symptoms such as fever, rash, involvement of the gastrointestinal tract, lungs, and nervous system. However, in Eleftheriou’s pediatric series of EGPA in the UK, none of the children tested positive for ANCA (15). Given the limited number of pediatric EGPA cases, it remains uncertain whether there is a correlation between clinical manifestations and ANCA in children.

The treatment of EGPA involves a multidisciplinary approach tailored to the specific needs of each patient. The main goals of therapy are to control inflammation, prevent organ damage, and improve quality of life. Systemic glucocorticoids continue to serve as the cornerstone of treatment, effectively suppressing inflammation and managing symptoms. Patients with severe disease require pulsed intravenous glucocorticoid treatment, typically administered as daily methylprednisolone pulses ranging from 500 to 1,000 mg each over a three-day period, with a maximum cumulative dose of 3 grams. Subsequently, high-dose oral glucocorticoids should be prescribed, such as 0.75 to 1 mg/kg per day, to maintain effective therapy (3). In severe cases, particularly when the central nervous system, heart, kidneys, or digestive tract are affected, immunosuppressive agents such as cyclophosphamide or methotrexate may be prescribed. These agents aim to modulate immune system activity and safeguard blood vessels and organs from further harm. For younger patients with involvement of the nervous system and myocardium, the use of high-dose intravenous immunoglobulin may be considered a viable alternative when standard treatment fails to produce satisfactory results. In select cases, biologic therapies, like rituximab, that target specific immune system components may also be explored (7, 16–19). For patients with relapsing-refractory disease without organ or life-threatening manifestations, interleukin-5 receptor antagonist in combination with steroids is recommended (3, 20, 21). Which can help reduce the need for oral glucocorticoids and potentially delay the progression of the disease (22–24). Currently, there are few cases of pediatric EGPA, and there is a lack of long-term follow-up data on the treatment of these patients. During the hospitalization, the child we reported had a rapidly progressing condition and multiple organs affected. Therefore, methylprednisolone in combination with CTX was chosen as the treatment option.

Suying Liu et al. discovered that individuals with damage to the CNS are more prone to experiencing digestive tract involvement (12). The microbiota-gut-brain axis theory offers a potential explanation for this connection (25). Cyclophosphamide is more effective at crossing the blood-brain barrier than rituximab. Therefore, glucocorticoids combined with cyclophosphamide are often the preferred initial treatment for EGPA affecting the CNS (26). Suying Liu et al. observed that intrathecal injection therapy had a positive impact. Zhou Jiaxin reported on 73 EGPA patients, finding that 11% had CNS involvement (27). All patients received methylprednisolone pulse therapy, and cyclophosphamide was the preferred treatment option.

In clinical practice, the five-factor score (FFS), which was meticulously developed and refined by the French Vasculitis Study Group, is utilized to assess disease risk factors and predict prognosis (1). It includes: (1) involvement of the gastrointestinal tract (2) cardiac involvement (3) serum creatinine levels surpassing 150 μmol/L (4) age exceeding 65 years (5) absence of ear, nose, and throat involvement. Each factor is assigned a score of 1, and higher cumulative scores indicate a poorer prognosis. However it fails to provide clear guidance for other adverse outcomes in EGPA patients, such as disability due to peripheral nerve involvement (20).

The case we are reporting is an EGPA patient with CNS who exhibits PRES accompanied by cerebral artery stenosis. Both domestically and internationally, there have been no reports on this condition in children. This highlights the diverse nature of neurological symptoms in EGPA patients. If patients exhibit allergic diseases, including asthma, allergic rhinitis, urticaria, and have elevated eosinophil counts, they should be highly suspected of having EGPA. It is crucial to conduct cranial imaging and other tests to diagnose EGPA as soon as possible and establish a corresponding treatment plan to improve the prognosis for these children.

EGPA is a rare condition, even more so in children. The absence of distinctive clinical manifestations makes it prone to misdiagnosis and delayed diagnosis, leading to many patients not receiving early diagnosis and treatment. Therefore, it is crucial to have a thorough understanding of the clinical characteristics of EGPA, diagnose and treat the condition promptly and consistently before severe organ involvement occurs, and prevent irreversible organ damage to enhance patient prognosis and quality of life.

## Data availability statement

The original contributions presented in the study are included in the article/supplementary material. Further inquiries can be directed to the corresponding author.

## Ethics statement

The studies involving humans were approved by the Affiliated Hospital of Qingdao University. The studies were conducted in accordance with the local legislation and institutional requirements. Written informed consent for participation in this study was provided by the participants’ legal guardians/next of kin. Written informed consent was obtained from the individual(s), and minor(s)’ legal guardian/next of kin, for the publication of any potentially identifiable images or data included in this article.

## Author contributions

NN: Conceptualization, Data curation, Formal Analysis, Writing – original draft. LL: Conceptualization, Data curation, Formal Analysis, Writing – original draft. CB: Data curation, Formal Analysis, Methodology, Writing – original draft. DW: Data curation, Supervision, Writing – original draft. SG: Data curation, Supervision, Writing – original draft. JL: Data curation, Writing – original draft. RZ: Formal Analysis, Investigation, Writing – original draft. YL: Data curation, Supervision, Writing – original draft. QZ: Data curation, Supervision, Writing – original draft. HC: Conceptualization, Investigation, Project administration, Writing – review & editing.
